# VOLUNTEERING, DISCRIMINATION, AND DEPRESSION IN OLD AGE: DOES THE FORM OF DISCRIMINATION PLAY A ROLE?

**DOI:** 10.1093/geroni/igad104.2883

**Published:** 2023-12-21

**Authors:** Huei-wern Shen

**Affiliations:** University of North Texas: Denton, Denton, Texas, United States

## Abstract

Older adults experiencing discrimination have an increased risk of poor mental health, such as depression. Discrimination could happen based on age, race, gender, sexual orientation, or other factors but the effects of experiencing different forms of discrimination on depression are unknown. Given the evidence that volunteering in old age promotes mental health, this study explores the relationship between volunteering and depressive symptoms among older adults who experience discrimination, and investigates whether such a relationship differs between those experiencing ageism and those experiencing other forms of discrimination. Using a subsample of respondents 65-96 years of age from the 2016 Health and Retirement Study, 1881 older adults who reported experiencing everyday discrimination were included. Discrimination was measured by the six-item Everyday Discrimination Scale. Among them, 955 respondents named age as the reason behind the discrimination experiences, and 926 named other forms (e.g. race). Controlling for health-related variables (e.g., ADLs/IADLs), SES (e.g., income), and sociodemographics (e.g., gender), findings from the weighted OLS regression models showed that volunteering (doing unpaid work for religious, educational, health-related, or other charitable organizations) related to lower depressive symptoms (8-item CES-D, P<.05). When looking at the groups who experienced different forms of discrimination, volunteering continued to have a negative relationship with depressive symptoms for older adults who experienced ageism (p<.01); however, such a relationship disappeared for the group experiencing other forms of discrimination. Discussions will target the importance of understanding the complicated relationships that may exist in volunteering, mental health, and the forms of discrimination that older adults experience.

